# Prevalence of Career Indecision and Factors Influencing It Among Medical Students and Interns in Oman: A Cross-Sectional Questionnaire Study

**DOI:** 10.7759/cureus.63953

**Published:** 2024-07-06

**Authors:** Ali Abdullah Al Ajmi, Fatma S Al Kharusi, Aisha H Al Khamisi

**Affiliations:** 1 Emergency Medicine, Oman Medical Specialty Board, Muscat, OMN; 2 Emergency Medicine, Sultan Qaboos University, Muscat, OMN

**Keywords:** career indecision, oman, medical interns, senior medical student, career factors inventory (cfi), prevalence

## Abstract

Aim and objectives: Career indecision is a broad term that refers to the uncertainty and difficulty of decision-making regarding future careers among junior professionals. This study aims primarily to estimate the prevalence of career indecisiveness among senior medical students and medical interns in Oman. Secondly, it assesses the association of sociodemographic factors influencing it. Finally, it examines the association between participation in career development activities and career indecision among them during the academic year of 2022-2023.

Methods: A cross-sectional study was conducted using an anonymous self-administered questionnaire in the English language. Unpaired t-test and ANOVA test were used to compare means between groups. A 21-item Career Factors Inventory (CFI) was used to determine the career indecision score. These scores were further classified as low-level (score 27-71) and high-level (score 72-105).

Results: The total number of participants was 161. The minimum sample size calculated was 153 participants for 95% confidence intervals. The prevalence of high-level career indecision was 63.4% (95%CI 55.4%-70.8%) among the participants. Participants with one of their parents in healthcare professions and those who did not participate in career development activities had high career indecision scores with P-values of 0.002 and 0.022, respectively. Moreover, participants younger than 25 years of age in comparison to older participants had higher need-for-self-knowledge (NSK) scores (p-value 0.018).

Conclusion: A high prevalence of high-level career indecision was seen among senior medical students and medical interns in Oman. Few factors were found to be statistically associated with career indecision, especially participation in career development activities. Further studies are recommended to investigate the causality of high-level career indecision among junior professionals in Oman and the contributing factors. Curricular and extra-curricular career development activities and counseling may reduce career indecision.

## Introduction

Career indecision is a broad term that refers to the uncertainty and difficulty during decision-making of the future career among junior professionals [1]. There are many papers that refer to this as career uncertainty [2-4]. However, the most encountered term in recent medical literature is "career indecision and indecisiveness" [5,6]. Career decision-making is a dynamic, progressive, complex, and multifactorial process, which occurs in many stages and includes the choice of a specialty, placement, and/or location of practice [7]. 

In medicine, students tend to use their clinical years as well as internship periods to refine their specialty and path preferences [3]. Hence, a few years are needed to have career crystallization [5]. However, difficulties like lack of readiness and resources are frequently experienced by medical students when making their career decisions [6]. Moreover, many studies showed that a significant proportion of final-year medical students had yet to commit to a specialty [3].

Prevalence of career indecision among medical students is estimated to be as high as 40% at entrance to medical school, 26% in junior students, and decreases to nearly 15% by graduation [2]. Despite the fact of career indecision resolution by years, there is evidence showing that it is not limited to medical school, but rather continues post graduation [2,3,7]. In fact, the prevalence of career indecision is morbid in many countries and is encountered yearly [5,8-12]. It is negatively associated with coping strategies and positively associated with psychological distress problems [6]. Moreover, it is described as a negative influence on peoples’ careers and should therefore be reduced or avoided [5,13]. Additionally, it is negatively associated with balanced medical workforce distribution [14,15].

The distribution of the medical workforce has been problematic for decades in many countries [14,16]. It has given rise to a wealth of studies examining the career decision-making process of medical students and junior medical professionals [7,16]. In countries with low doctor-population ratios, there is a need for equitable distribution of the healthcare workforce for a better national healthcare system [14,16]. 

While factors and predictors behind choosing a specialty and being certain about a medical career path have been studied extensively in nearly all medical specialties, worldwide, there is little data in literature exploring the tangible factors behind career indecision. Few factors are proposed to have been associated with career indecision in literature including poor career guidance and resources, lack of readiness, young age, and the female gender [5,14,15]. 

A study done in Malaysia in 2021 showed that 99% of students had a high level of career indecision [5]. In 2018, a study in Saudi Arabia that aimed to study factors and predictors of choosing Emergency Medicine as a specialty among graduating medical students, showed indirectly a prevalence of 6% of undecided students [17]. In 2012, a study in Kuwait aimed to explore the specialty of interest among medical students of different levels [8]. Noticeably, more than 50% of the students in clinical years of medical school had not decided what specialty to pursue.

While prevalence and factors differ greatly between countries and the medical literature is yet exploring them, this research is exploring the prevalence of career indecision and factors influencing it locally [18]. Examining the country-based prevalence of career indecision and the factors influencing it among senior medical students and medical interns will help to reduce the burden of it, and plan and determine the future composition of the physician workforce in Oman [1,6,10-12,19]. 

This study aimed primarily to estimate the prevalence of career indecision among senior medical students and medical interns in Oman. Secondly, it assesses the association of sociodemographic factors and career indecision among the participants. Finally, it examines the association between participation in career development activities and career indecision among them.

## Materials and methods

This was a cross-sectional study conducted at the College of Medicine and Health Sciences, Sultan Qaboos University (SQU), and the College of Medicine, National University for Sciences and Technology (NUST), in Oman. Only senior medical students and medical interns were included in the study. The study was conducted from December 2022 to March 2023, corresponding with the residency program application period for the Oman Medical Specialty Board (OMSB). This period was chosen because it matches the largest possible number of senior medical students and medical interns in Oman. Ethical approval for the study was acquired from the Medical Research Ethics Committee, College of Medicine and Health Sciences, Sultan Qaboos University (approval number: MREC#2877). All participation was voluntary and informed consent was gained before using any data. Surveys were archived anonymously and confidentially.

Sample size

The sample size was calculated using sample size for frequency in a population, available online through Open EpI (www.openepi.com). The total population was calculated using data from the College of Medicines of both SQU and NUST, which estimated a total number of approximately 400 senior medical students and medical interns. An estimated prevalence of 20% was considered based on the literature [2,5,9,17,18]. The minimum sample size calculated was 153 participants for a 95% confidence interval. We approached all eligible candidates for participation using official college emails and volunteers among them taking into consideration the best response rate.

Data collection

Data was collected using an anonymous self-administered questionnaire in the English language (See Appendices). The questionnaire was distributed online using Google Forms (Google LLC, Mountain View, California, United States). It was divided into three sections. The first section included an introduction to the study and the consent to participate. The second section collected independent factors of sociodemographic data including age, gender, nationality, level of career (senior medical student or medical intern), monthly family income, parents’ occupation (whether in healthcare or not), and participation in career development activities. Finally, the third section covered the dependent factors that determine the career indecisiveness score using the 21-item Career Factors Inventory (CFI) questionnaire.

The CFI questionnaire is validated, used, and published in literature internationally [5,20-22]. Formerly, the CFI questionnaire showed test-retest reliabilities ranging from 76% to 94% [20]. Internal consistency reliabilities ranged from 73% to 91% for the four subscales and from 73% to 92% for the total inventory [20]. The questionnaire consists of four subscales: Career Choice Anxiety (CCA, items 1-6), Generalized Indecisiveness (GI, items 7-11), Need for Career Information (NCI, items 12-17), and Need for Self-Knowledge (NSK, items 18-21). Permission for using the questionnaire was acquired via e-mail in July 2022. It is available online [5,20].

Responses were collected using a Likert scale of five graded scores ranging from 1 (lowest score) to 5 (highest score) per item of 21-item CFI. Career Indecisiveness score was calculated by summing up the score of all the CFI components. According to recent literature, the score was then classified as low-level career indecisiveness (score 27-71) and high-level career indecisiveness (score 72-105) [15].

Data analysis

Data analysis was done using IBM SPSS Statistics for Windows, Version 29.0 (Released 2022; IBM Corp., Armonk, New York, United States). An experienced statistician ran all statistical tests. Mean and standard deviation (SD) were used to show the results. Unpaired t-test and ANOVA test were used to compare between study groups. A significant difference was determined when the calculated p-value was <0.05 with a confidence interval of 95% after ensuring a response rate of at least 80% of the calculated sample size.

## Results

The total number of participants was 161, which was divided into two training levels. Senior medical students consisted of 63.4% of the study population with 102 participants and there were 59 (63.4%) medical interns (Table 1).

**Table 1:** Sociodemographic details and frequencies of participants (N=161).

Variable	Frequency (Percentage)
Training Level	
Senior medical student	102 (63.4)
Medical intern	59 (36.6)
Medical College	
Sultan Qaboos University (SQU)	110 (68.3)
National University for Science and Technology (NUST)	43 (26.7)
Abroad	8 (5.0)
Age Group	
<25 years	94 (58.4)
>=25 years	67 (41.6)
Sex	
Male	39 (24.2)
Female	122 (75.8)
Nationality	
Omani	155 (96.3)
Non-Omani	6 (3.7)
Monthly family income in Omani Rial (OMR)	
<1000 OMR	42 (26.1)
1000-2000 OMR	58 (36.0)
2000-3000 OMR	29 (18.0)
>3000 OMR	32 (19.9)
Parents’ Occupation	
Both in medical/healthcare profession	4 (2.5)
One in medical/healthcare profession	13 (8.1)
Neither in medical/healthcare profession	144 (89.4)

**Figure 1:**
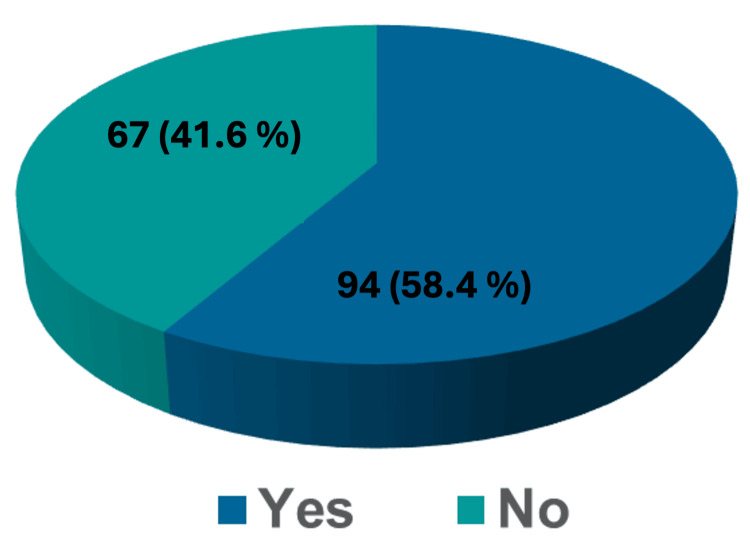
Previous participation in career development activities

Regarding the previous participation in career development activities (Figure 1), 94 (58.4%) had participated in such activities, while the remaining 67 (41.6%) had not. 

Career Indecisiveness score was calculated by summing up all the CFI components. The study revealed a prevalence of high-level career indecision equaling to 63.4% (95%CI 55.4%-70.8%). However, only 59 (36.6%) showed low-level career indecision (Table 2). The minimum score was 41 in contrast to the maximum score of 99. Table 3 shows the mean and standard deviation of career indecision and each subscale. In this study, the career indecision mean score was 74.9. 

**Table 2:** Level of career indecisiveness among participants (N=161)

Variable	Frequency (Percentage)
Low-level career indecisiveness (27-71)	59 (36.6)
High-level career indecisiveness (72-105)	102 (63.4)

**Table 3:** Mean and standard deviation of career indecisiveness scales among participants (N=161)

Variable	Mean±SD
Career Indecisiveness Score	74.9±10.97
Career Choice Anxiety	18.8±3.94
Generalized Indecisiveness	15.4±4.58
Need for Career Information	24.1±4.55
Need for Self-Knowledge	16.5±3.28

Table 4 shows the unpaired t-test and ANOVA test for the association between sociodemographic factors and career indecision. Participants with one of their parents in healthcare professions and those who did not participate in career development activities had high career indecision scores with p-values of 0.002 and 0.022, respectively. Moreover, participants younger than 25 years of age had higher NSK scores in comparison to older participants (p-value=0.018). Other tested sociodemographic factors were found to have no statistically significant difference.

**Table 4:** Unpaired t-test and ANOVA test for association between sociodemographic factors and career indecision (N = 161) *Group 1: Both in Medical/Healthcare profession; **Group 2: One in Medical/Healthcare profession; ***Group 3: Neither in Medical/Healthcare profession. OMR: Omani Rial; SQU: Sultan Qaboos University; NUST: National University for Science and Technology

Socio-demographic factors		Career Indecisiveness Score		Career Choice Anxiety		Generalized Indecisiveness		Need for Career Information		Need for Self-Knowledge	
		Mean±SD	p-value	Mean±SD	p-value	Mean±SD	p-value	Mean±SD	p-value	Mean±SD	p-value
Training Level	Senior medical student	74.3±11.27	0.32	18.6±4.02	0.3	15.1±4.53	0.18	24.0±4.89	0.71	16.6±3.58	0.77
	Medical intern	76.1±10.42		19.3±3.79		16.1±4.63		24.3±3.93		16.4±2.73	
Medical College	SQU	75.2±10.28	0.8	18.7±3.94	0.72	15.7±4.73	0.22	24.0±4.43	0.84	16.9±3.01	0.1
	NUST	74.0±13.32		19.0±4.08		14.5±4.27		24.4±5.14		16.0±3.82	
	Abroad	75.6±5.88		19.9±3.40		17.1±3.52		23.9±2.70		14.8±3.20	
Age Group	<25 years	75.2±11.45	0.67	18.5±3.85	0.23	15.4±5.50	0.99	24.2±4.83	0.69	17.1±3.22	0.018
	>=25 years	74.5±10.33		19.3±4.04		15.4±4.73		23.9±4.16		15.8±3.25	
Sex	Male	73.8±10.81	0.48	18.2±4.21	0.23	14.6±4.60	0.20	24.9±4.14	0.19	16.1±3.27	0.34
	Female	75.3±11.04		19.1±3.84		15.7±4.56		23.8±4.65		16.7±3.29	
Monthly Family Income	<1000 OMR	74.8±9.11	1.00	18.6±3.42	0.84	14.8±4.18	0.80	24.5±3.96	0.7	16.8±3.04	0.67
	1000-2000 OMR	74.9±12.16		18.8±3.51		15.6±4.65		23.9±5.37		16.5±3.56	
	2000-3000 OMR	74.9±10.93		18.5±4.94		15.8±5.75		24.6±3.79		15.9±3.36	
	>3000 OMR	75.1±11.48		19.4±4.41		15.5±3.82		23.4±4.36		16.8±3.05	
Parents’ Occupation	Group 1*	68.3±3.59	0.002	18.3±0.5	0.61	13.8±3.40	0.042	21.0±4.24	0.015	15.3±2.75	0.005
	Group 2**	84.8±8.49		19.9±3.96		18.4±5.12		27.2±2.52		19.3±0.95	
	Group 3****	74.2±10.85		18.8±3.99		15.2±4.48		23.9±4.59		16.3±3.32	

Table 5 shows the association between previous participation in career development activities and career indecision using unpaired t test. For the career indecision score, 72.6±9.94 participants had previous participation in career development activities in comparison with 76.6±11.41 who did not have previous participation (p-value 0.022); hence, there was a statistically significant difference. 

**Table 5:** Unpaired t-test for association between previous participation in career development activities and career indecision (N = 161) No: No previous participation in career development activities; Yes: Previous participation in career development activities *significant difference

Variable		Mean±SD	p-value
Career indecisiveness Score	No	76.6±11.41	0.022*
	Yes	72.6±9.94	
Career Choice Anxiety	No	19.6±3.77	0.004*
	Yes	17.8±3.95	
Generalized Indecisiveness	No	16.4±4.57	0.002*
	Yes	14.1±4.30	
Need for Career Information	No	24.1±4.81	0.951
	Yes	24.1±4.19	
Need for Self-Knowledge	No	16.5±3.51	0.992
	Yes	16.5±2.95	

## Discussion

The primary objective of the study was to estimate the prevalence of career indecision among senior medical students and medical interns in Oman. The secondary objectives included assessing the association between socio-demographic factors and career indecision and examining the association between participation in career development activities and career indecision among them. 

The study included 161 participants, senior medical students represented approximately two-thirds of the study population which can be explained by the fact that senior medical students were approached through institutional emails while medical interns received the invitation through personal email. Moreover, medical students might have been more interested in participation, especially when future and career were discussed. Medical interns who have already decided their career path and future might have been less concerned; hence participation may have been low. 

The study results showed that participants had poor participation in career development activities as only 58.4% of them attended such activities. This raises a concern regarding both curricular and extra-curricular activities organized during medical school and internship programs. However, knowing that only less than half of the participants had not participated in such activities, this could be explained by many reasons including poor attendance, low number of conducted career development activities, and lack of interest. 

The prevalence of career indecision can be estimated using quantitative and qualitative methodologies. This study used the CFI score, which is a quantitative method to estimate career indecision. A high-level career indecisiveness score reflects career indecision while a low career indecisiveness score is considered to be healthy. The prevalence of career indecision in our setting is 63.4%. A recent study in 2021 done in Malaysia, showed 99% of their students had a high level of career indecisiveness [5]. In the above-mentioned study, the high prevalence was explained by many factors including the COVID-19 pandemic due to diversion to online classes and compromised clinical exposure, and a third of the study population were pre-clinical students. In contrast, our study was conducted after the COVID-19 pandemic and the targeted populations were senior medical students and medical interns. 

The prevalence found in the current study is in keeping with a study in Kuwait prior to the COVID-19 pandemic in 2012, in which more than 50% of the students in clinical years of medical school were undecided about what specialty to pursue [18]. There are limited studies in the literature exploring the prevalence of career indecision among junior medical professionals, i.e. graduating medical students and medical interns. Moreover, the settings of the study population and medical background are different which makes local studies necessary to identify the prevalence and explore influencing factors.

Unexpectedly, there is no statistically significant difference regarding career indecision in terms of training between senior medical students and medical interns. Additionally, there is no significant difference in CFI sub-scales. In literature, it is known that career indecision decreases by advancing training level [2,9]. Our findings could not be explained by the aforementioned literature. A number of factors could be responsible for this discrepancy, including the quality of career guidance activities in internship programs, limited exposure to various specialties, limited advanced subspecialty rotations, limited elective rotations, and possibly similar career guidance across both groups [23]. There is no statistical difference between medical schools in career indecision scores, which reduced the chance of confounding error. 

Young age and female sex are mentioned in the literature as factors that influence career indecision [5,14,15]. According to our results, we found that participants who are younger than 25 years of age have significantly higher NSK. Our results support recent literature [5,24]. In Oman, there is no gender discrimination which has likely prevented any influence of sex on career indecision. Although one would argue regarding socioeconomic influence on career indecision, according to current results, the monthly family income had not influenced career indecision (p-value 1.00). 

Parents’ occupations influence career decisions through motivation, guidance, and role model effects. It is no surprise that participants with both parents who are either medical or healthcare professionals have the lowest career indecision scores. On the other hand, when neither parent is practicing a healthcare profession, there is a high career indecision score. In contrast, a recent study in 2021 done in Malaysia didn’t find such influence [5]. Even according to the results of the current study, this is not fully applicable as results show that participants with one parent in the medical or healthcare profession have the highest career indecision scores and there is a statistically significant difference. This phenomenon has been explored in career aspirations and the influence of parents on their children’s career choices, with findings that maternal influence may be "diametrically opposed" to paternal influences [25,26].

The association between previous participation in career development activities and career indecision has been highlighted many times. Indeed, the necessity of career development activities for junior professionals and students is universally acknowledged and undeniable. This is strongly supported by a systematic review by Priyashantha et al. [27]. 

Our study findings suggest an utmost need to promote earlier career awareness and development programs to reduce the burden of career indecision among junior professionals. An internship program can be modified to allow more career development curriculum and activities including elective rotations and counseling. There are a number of adjustable factors that can be considered and used by stakeholders and medical education bodies in Oman to refine the needs and better understand local improvement areas. This study opens the floor for further studies that can be done to explore other factors affecting career indecision like mental health status, academic performance, clinical rotation exposure, and clinical electives.

Given the nature of this study, we have to acknowledge certain limitations that future studies should aim to minimize. Firstly, the study itself is based on a self-administered online questionnaire that was conducted without face-to-face explanation, and thus there could be potential for bias. Secondly, as the majority of respondents (63.4%) were senior medical students while only 36.6% were medical interns, this could have affected our outcome. Finally, we could not study the changes in career indecision scores over time among them and their progress.

## Conclusions

Study participants showed a high prevalence of high-level career indecision among senior medical students and medical interns in Oman. Few factors were found to be statistically associated, especially participation in career development activities. Further studies are recommended to investigate the causality of high career indecision levels among junior professionals in Oman and the contributing factors. We recommend doing more studies to assess causality and to test other factors. Curricular and extra-curricular career development activities and counseling may reduce career indecision and its burden among junior professionals.

## Appendices

Questionnaire

A. Section One: Introduction and Consent

Dear participants, this survey is aimed to be used for data collection for a study titled: Prevalence of Career Indecision and Factors Influencing It Among Medical Students and Interns in Oman: A Cross-Sectional Questionnaire Study. It is ethically approved by the Medical Research Ethics Committee (MREC) at the College of Medicine & Health Science, SQU.

Career indecision is a broad term that refers to the uncertainty and difficulty during decision-making of the future career among junior professionals. In fact, the prevalence of career indecision is morbid in many countries and is encountered yearly. A few factors are proposed to have been associated with career indecision in literature including poor career guidance and resources, lack of readiness, and being young along with female gender. Hence, this study is conducted to estimate the prevalence of career indecision and factors influencing it among senior medical students and medical interns in Oman during the academic year of 2022-2023. This study targets all senior medical students and medical interns in Oman. Data will be used anonymously by the project team for analysis and results will be published. We disclose that there is no conflict of interest.

* Indicates required question

1. Do you consent to participate in this study?*

Yes

No

2. You can ask to be withdrawn from the study at any time with no reason. You will be asked to provide the following email to reach your data for withdrawal. (Optional)

B. Section Two: Socio-Demographic Data

1. You are*

Senior medical student

Medical intern

2. You are studying/ studied medicine in*

3. Age group*

<25 years

25 years and more

4. Gender*

Male

Female

5. Nationality*

Omani

Other

6. Monthly family income in Omani Riyals (OMR)*

<1000

1000-2000

2000-3000

>3000

7. Parents' occupation*

Both in Medical / Healthcare profession

One in Medical / Healthcare profession

None in Medical / Healthcare profession

8. Participated in career development activities*

Yes

No

C. Section Three: Career Factors Inventory (CFI)

I. Career Choice Anxiety (CCA): When you start thinking about your future medical career-path.

Description of section

*Very low scores are indicative of abnormally no stress or ignorance. Mild-moderate scores indicates healthy stress. High scores indicates high level of anxiety during career-choice process*.

1. When I think about actually deciding what I want my future medical career to be I feel:*

Select from 1 (Fearless) to 5 (Frightened)

2. When I think about actually deciding what I want my future medical career to be I feel:*

Select from 1 (Relaxed) to 5 (Tensed)

3. When I think about actually deciding what I want my future medical career to be I feel:*

Select from 1 (Carefree) to 5 (Worried)

4. When I think about actually deciding what I want my future medical career to be I feel:*

Select from 1 (Calm) to 5 (Jittery)

C. Section Three: Career Factors Inventory (CFI)

II. Generalized Indecisiveness (GI): your certainty in decisions.

Description of section

This section tests your general process of decision-making, especially in career choices.

1. For me, the decision seems*

Select from 1 (Easy) to 5 (Hard)

2. For me, decision-making seems*

Select from 1 (Clear) to 5 (Hazy)

3. For me, decision-making seems*

Select from 1 (Fulfilling) to 5 (Frustrating)

4. When making most decisions I am*

Select from 1 (Quick) to 5 (Slow)

5. When making most decisions I am*

Select from 1 (Certain) to 5 (Uncertain)

C. Section Three: Career Factors Inventory (CFI)

III. Need for career information (NCI)

Description of section

This section will provide information about the need to have detailed information using time, practice, and other resources before making a career decision.

1. Before choosing or entering a particular career area I still need to talk to people in one or more various medical careers*

Select from 1 (Strongly disagree) to 5 (Strongly agree)

2. Before choosing or entering a particular career area, I still need to gain practical knowledge of different specialties through as many part-time and summer electives/rotations as possible.*

Select from 1 (Strongly disagree) to 5 (Strongly agree)

3. Before choosing or entering a particular career area, I still need to find out what are the present and predicted job opportunities like for a certain career area or areas.*

Select from 1 (Strongly disagree) to 5 (Strongly agree)

4. Before choosing or entering a particular career area I still need to use my free time and school courses to help determine what type of career I might enjoy and do well in.*

Select from 1 (Strongly disagree) to 5 (Strongly agree)

5. Before choosing or entering a particular career area I still need to familiarize myself with one or a number of college majors and their requirements.

Select from 1 (Strongly disagree) to 5 (Strongly agree)

6. Before choosing or entering a particular career area I still need to familiarize myself with one or a number of specialties and their requirements.*

Select from 1 (Strongly disagree) to 5 (Strongly agree)

C. Section Three: Career Factors Inventory (CFI)

IV. Need for self-knowledge (NSK)

Description of section

This section will provide us with information about your need to have more awareness and self-knowledge, particularly in determining the career choice and decision process.

1. Before choosing or entering a particular career area I still need to attempt to answer "who am I?"*

Select from 1 (Strongly disagree) to 5 (Strongly agree)

2. Before choosing or entering a particular career area I still need to attempt to answer "what are my personal values?"*

Select from 1 (Strongly disagree) to 5 (Strongly agree)

3. Before choosing or entering a particular career area I still need to attempt to answer "what type of person would I like to be?*

Select from 1 (Strongly disagree) to 5 (Strongly agree)

4. Before choosing or entering a particular career area I still need to attempt to answer "what things are the most important to me?"*

Select from 1 (Strongly disagree) to 5 (Strongly agree)
